# Allotriophagy in a Patient With Schizophrenia: A Case Report

**DOI:** 10.7759/cureus.86375

**Published:** 2025-06-19

**Authors:** Sarah B Guttman, Mateus R Alessi, Enzo S Carioni, Bruna Furukawa, Valentina G Ramos, Gabriella Shinmi, Carlos Naufel, Alexandre K Mousfi, Sivan Mauer

**Affiliations:** 1 Medicine, Universidade Positivo, Curitiba, BRA; 2 Surgery, Mackenzie Evangelical University Hospital, Curitiba, BRA; 3 Medicine, Evangelical Mackenzie Faculty of Parana, Curitiba, BRA; 4 Medicine, Mackenzie Evangelical University Hospital, Curitiba, BRA; 5 General Surgery, Mackenzie Evangelical University Hospital, Curitiba, BRA; 6 Psychiatry, Mackenzie Evangelical University Hospital, Curitiba, BRA; 7 Psychiatry, Evangelical Mackenzie Faculty of Parana, Curitiba, BRA; 8 Psychiatry, Tufts University Medical School, Boston, USA

**Keywords:** eating disorders, emergency psychiatry, pica, psychiatry, schizophrenia

## Abstract

Allotriophagy, also known as pica, is an eating disorder characterized by the recurrent and compulsive ingestion of non-nutritive substances, such as metals, glass, and paper. This behavior may be associated with various medical and psychiatric conditions, being frequently reported in patients with schizophrenia. Schizophrenia is a chronic mental disorder that impairs an individual’s cognitive and behavioral functioning, potentially leading to heightened impulsivity and risky behaviors, such as the ingestion of foreign objects. This case report aims to describe an episode of allotriophagy in a schizophrenic patient, discussing its clinical and therapeutic implications. The patient, a male with a prior diagnosis of schizophrenia, exhibited recurrent episodes of object ingestion, including pens, toothbrushes, cardiac monitoring electrodes, and glass fragments. During hospital admission, surgical interventions were performed to remove the foreign bodies, alongside adjustments to psychiatric treatment with antipsychotics and mood stabilizers. Despite these measures, the patient persisted in compulsive ingestion behavior, requiring multiple hospital readmissions. This case highlights the complexity of the clinical and psychiatric management of allotriophagy associated with schizophrenia, emphasizing the need for multidisciplinary therapeutic approaches. Continuous reassessment of psychiatric treatment, psychosocial support, and containment strategies are essential to minimize risks and reduce the recurrence of this behavior. It is concluded that allotriophagy in schizophrenic patients poses a significant challenge to medical practice, necessitating integrated protocols that combine clinical monitoring, effective pharmacological interventions, and ongoing social support. Furthermore, the scarcity of longitudinal studies on the relationship between allotriophagy and schizophrenia underscores the need for further research to explore therapeutic alternatives and preventive strategies.

## Introduction

Allotriophagy, or pica, is an eating disorder characterized by the compulsive ingestion of non-nutritive substances, such as soil, paper, glass, and metals. It is frequently associated with psychotic disorders, particularly schizophrenia, though it also occurs in other medical and psychiatric conditions. The health risks vary depending on the ingested material and include gastrointestinal obstructions, perforations, intoxications, malnutrition, and infections, which can be potentially fatal. Ingestion of heavy metals, such as lead, may cause severe systemic poisoning, affecting organs such as the brain, liver, and kidneys, while the ingestion of sharp objects increases the risk of digestive hemorrhage and peritonitis, requiring urgent medical intervention [1].

Schizophrenia is a chronic, psychotic, progressive, and debilitating psychiatric disorder that affects an individual’s perception, thinking, and behavior. Patients with schizophrenia may exhibit cognitive impairments and impulsivity, factors that contribute to atypical behaviors such as allotriophagy [2]. The hyperdopaminergic hypothesis is associated with the positive symptoms of schizophrenia, which include distortions or exacerbations of normal functions such as delusions and hallucinations. In contrast, the hypoglutamatergic hypothesis is linked to the negative symptoms, which involve reductions or losses of normal functions such as apathy and social isolation. Neuroimaging studies indicate a reduction in white matter volume in areas such as the prefrontal cortex and subcortical regions, impairing essential cognitive functions. These structural changes, present from the early stages of the disease, suggest widespread dysfunction in brain connectivity, with failures in the differentiation and maturation of glial cells, resulting in progressive functional impairment and structural disarray [3].

The prevalence of allotriophagy in patients with schizophrenia is higher than in the general population, possibly due to the interplay of impulsivity, cognitive alterations, and nutritional deficiencies. In the general population, it is estimated to range between 8% and 65%, depending on age group, socioeconomic conditions, and clinical context. Among patients with schizophrenia, the rate reaches 14.3%, compared to only 1.5% in the general population. Furthermore, the pathophysiology of schizophrenia, marked by dopaminergic and glutamatergic dysregulation, may contribute to compulsive and risky behaviors, such as the ingestion of non-nutritive substances [4].

The clinical management of allotriophagy involves multiple approaches. Removal of foreign bodies may require endoscopic or surgical procedures, depending on the size and location of the ingested object (1). Psychiatric treatment should be optimized with the use of antipsychotics, particularly second-generation ones, which have shown reductions in impulsivity and associated obsessive-compulsive symptoms [2]. In some cases, psychotherapy and sociotherapy are also recommended to reduce the recurrence of the behavior [2].

Moreover, the psychosocial impact of allotriophagy in patients with schizophrenia cannot be underestimated. The condition can lead to social stigma, family difficulties, and limitations in daily activities, significantly affecting the quality of life of both patients and their caregivers. Psychosocial support strategies, such as support groups and family education, are essential to improve clinical outcomes and promote social reintegration [5].

Despite advances in understanding allotriophagy, there remains a scarcity of longitudinal studies and randomized clinical trials evaluating prevention and treatment strategies. Future research should focus on developing integrated protocols that address both the psychiatric and medical aspects of allotriophagy, aiming to improve clinical outcomes and patients’ quality of life.

The scarcity of case reports in the literature on allotriophagy in schizophrenia underscores the importance of documenting and disseminating such occurrences. This study contributes to this goal by describing an atypical case of allotriophagy, highlighting its complications and clinical implications for the management of psychiatric patients.

## Case presentation

A 33-year-old male patient with a diagnosis of schizophrenia and a history of auditory hallucinations, impulsivity, and foreign body ingestion was admitted to the hospital on January 17, 2025. He was referred from the Emergency Care Unit, restrained on a rigid stretcher, after reporting the ingestion of a ballpoint pen and two intravenous catheters. His medical history included multiple psychiatric hospitalizations and an exploratory laparotomy four months prior for the removal of eight toothbrushes. He was on clozapine 100 mg/day, lithium carbonate 300 mg at night, valproic acid 500 mg twice daily, and diazepam 10 mg at night, with a history of heavy smoking and crack use since age 12.

**Figure 1:**
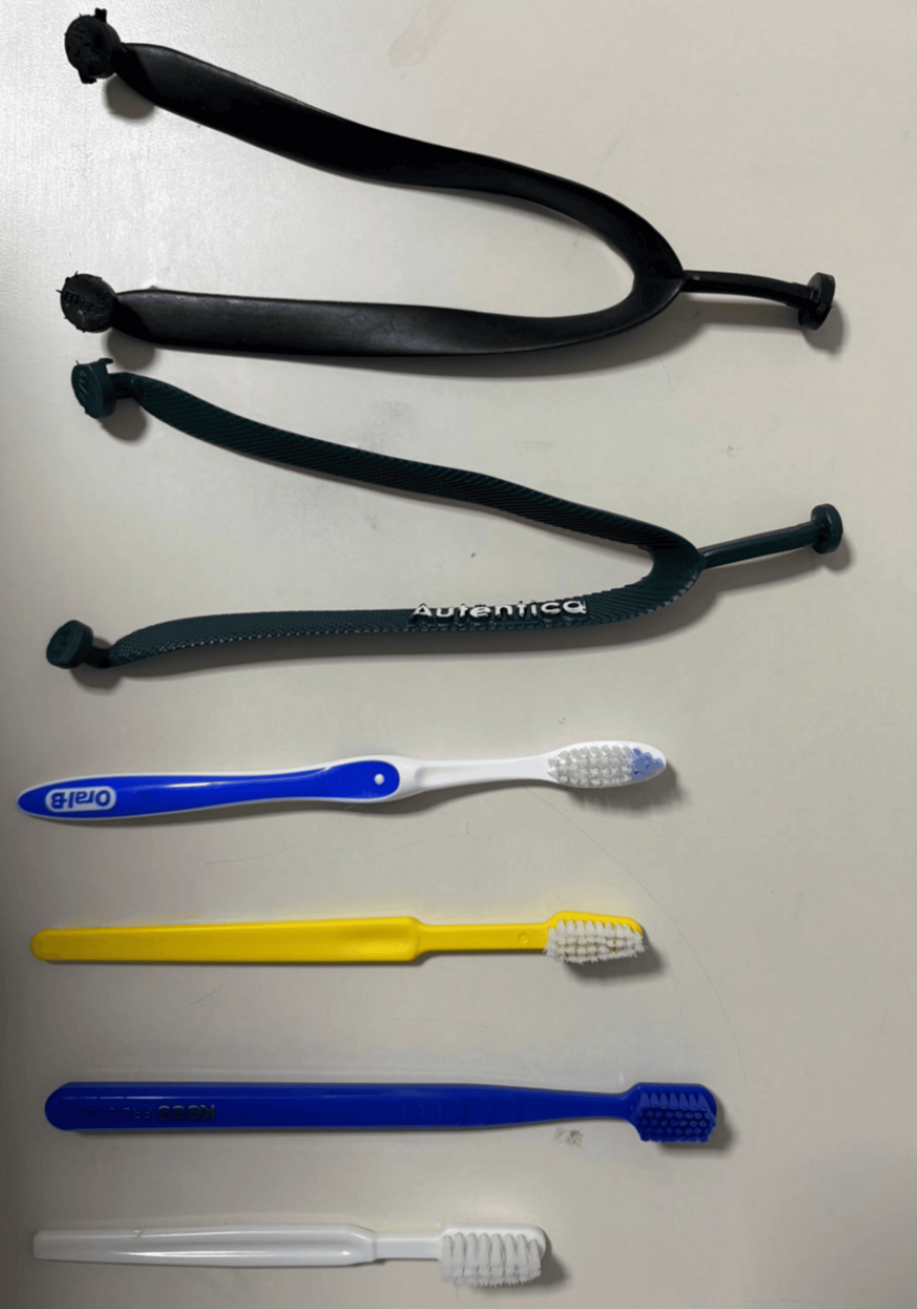
Foreign objects ingested by the patient, removed through upper digestive endoscopy (UDE). The image shows the foreign objects ingested by the patient and retrieved during an upper digestive endoscopy (UDE). The items were successfully extracted endoscopically and are presented here for documentation purposes. At the treating institution, images from the UDE are not archived in the hospital system; only the written procedural report is retained.

Upon admission, the patient was hemodynamically stable, with no significant gastrointestinal complaints. No acute psychiatric symptoms were noted at the time of evaluation. Physical examination revealed no signs of peritonitis. Imaging studies and upper digestive endoscopy (UDE) were conducted, confirming the presence of foreign bodies in the stomach (Figure 1). However, complete endoscopic removal was not feasible. Based on these findings, an exploratory laparotomy was indicated and performed on January 17, 2025. Intraoperatively, a pen was removed from the stomach and an intravenous catheter from the jejunum, without complications. The abdominal cavity inventory showed normal intestinal loops with no signs of perforation or infection.

In the following days, the patient remained hospitalized under observation, with no gastrointestinal complaints and good dietary tolerance. However, he exhibited episodes of psychomotor agitation, escape attempts, and further ingestion of objects, including three cardiac monitoring electrodes and an oximeter, despite mechanical restraint. The medical team reinforced restraints and increased the dose of dexmedetomidine hydrochloride for continuous infusion (0.2-0.7 mcg/kg/h, as clinically needed) to manage agitation.

On January 20, a psychiatric evaluation reinforced the diagnosis of schizophrenia with auditory hallucinations and suicidal ideation. The patient was maintained on antipsychotic and mood-stabilizing medications, adjusted to valproic acid 500 mg at night, risperidone 2 mg at night, and lithium carbonate 300 mg at night. If oral administration was not possible, haloperidol 5 mg intramuscularly every 12 hours was indicated as needed until oral intake could resume.

On January 21, he ingested three monitoring electrodes and an oximeter, necessitating an urgent UDE. Haloperidol, promethazine, and midazolam were administered intramuscularly. Treatment was adjusted to valproic acid 1,000 mg daily (500 mg morning and 500 mg night), clozapine 100 mg at night, lithium 900 mg at night, diazepam 10 mg daily (5 mg morning and 5 mg night), and risperidone 4 mg daily (2 mg morning and 2 mg night).

Throughout the hospitalization, the patient reported feelings of abandonment, emphasizing the absence of his sister, his only family connection. He expressed distress regarding past psychiatric hospitalizations, stating a preference for transfer to a clinic where he had a more positive experience.

On January 29, 2025, he verbalized active suicidal ideation, requested psychological care, and asked for mechanical restraint due to fear of self-harm. The social work team contacted his family, who reported being unable to visit due to recent motherhood and cited past family conflicts, including episodes requiring police intervention. After discussion with the psychiatric team, the patient was registered with the Psychiatric Bed Registry and transferred to a clinic on January 31.

**Figure 2:**
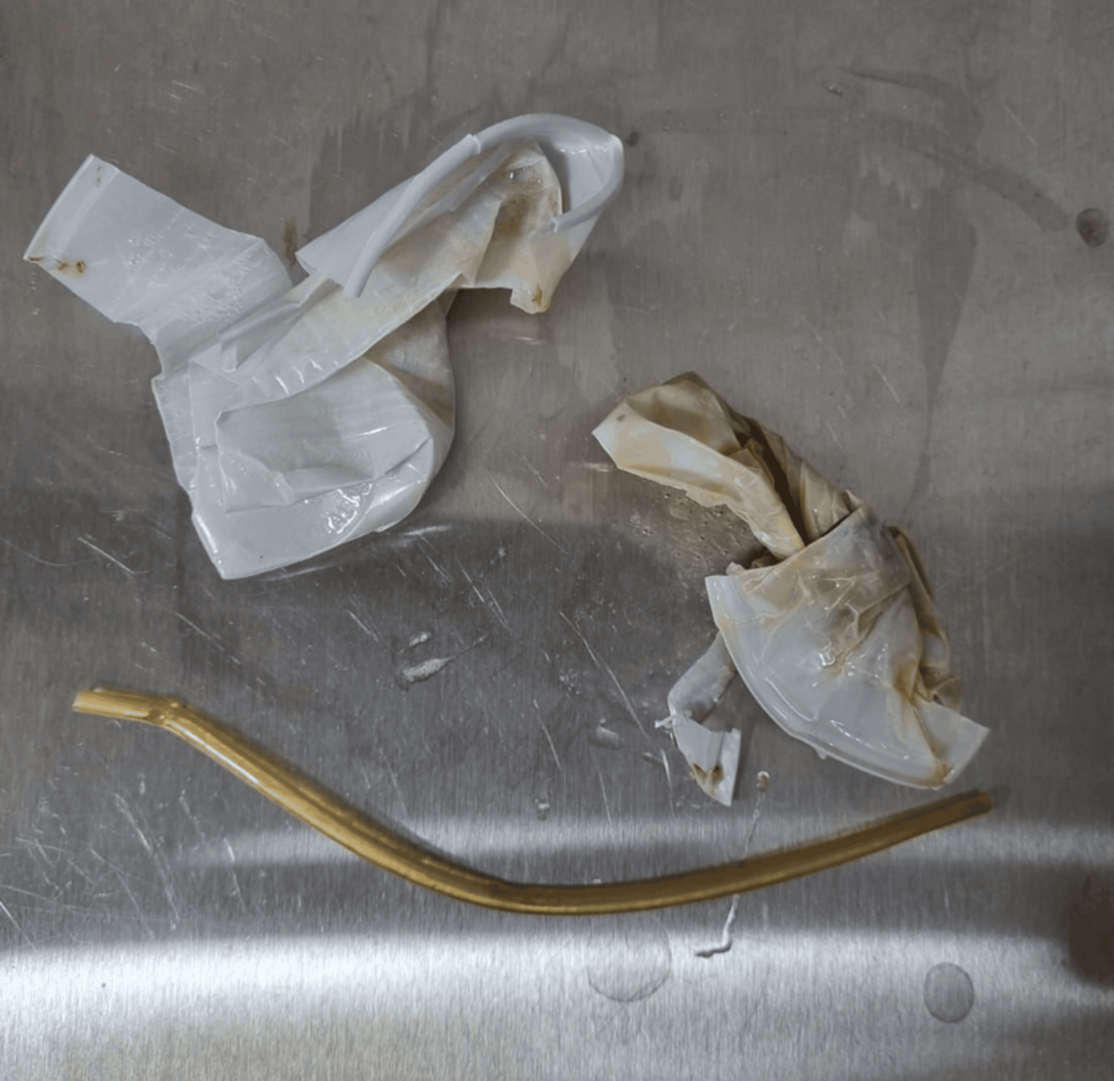
Plastic cups ingested by the patient. Plastic cups ingested by the patient, which were successfully identified and removed through an upper digestive endoscopy (UDE), as evidenced by the photographic documentation captured during the procedure. This image serves to illustrate the nature and size of the foreign bodies extracted, providing valuable support for clinical evaluation and ongoing medical management of the case.

However, on February 1, he was readmitted to the hospital after ingesting two plastic cups (Figure 2) and, the next day, glass fragments from a window. He reported moderate pain and vomiting with a small amount of blood. An urgent endoscopy removed three foreign bodies from the stomach, with no evidence of severe injury. After stabilization, he was transferred back to the psychiatric clinic on February 3 but returned on February 6 with suspected surgical suture dehiscence. Evaluation, however, showed no surgical complications, allowing his return to the clinic.

On March 11, 2025, he was referred to the hospital again after ingesting a toothbrush. He reported moderate epigastric pain, with no signs of severe complications. Endoscopy confirmed the object in the stomach, which was removed with an endoscopic loop without incident. He was discharged with instructions on warning signs.

**Figure 3:**
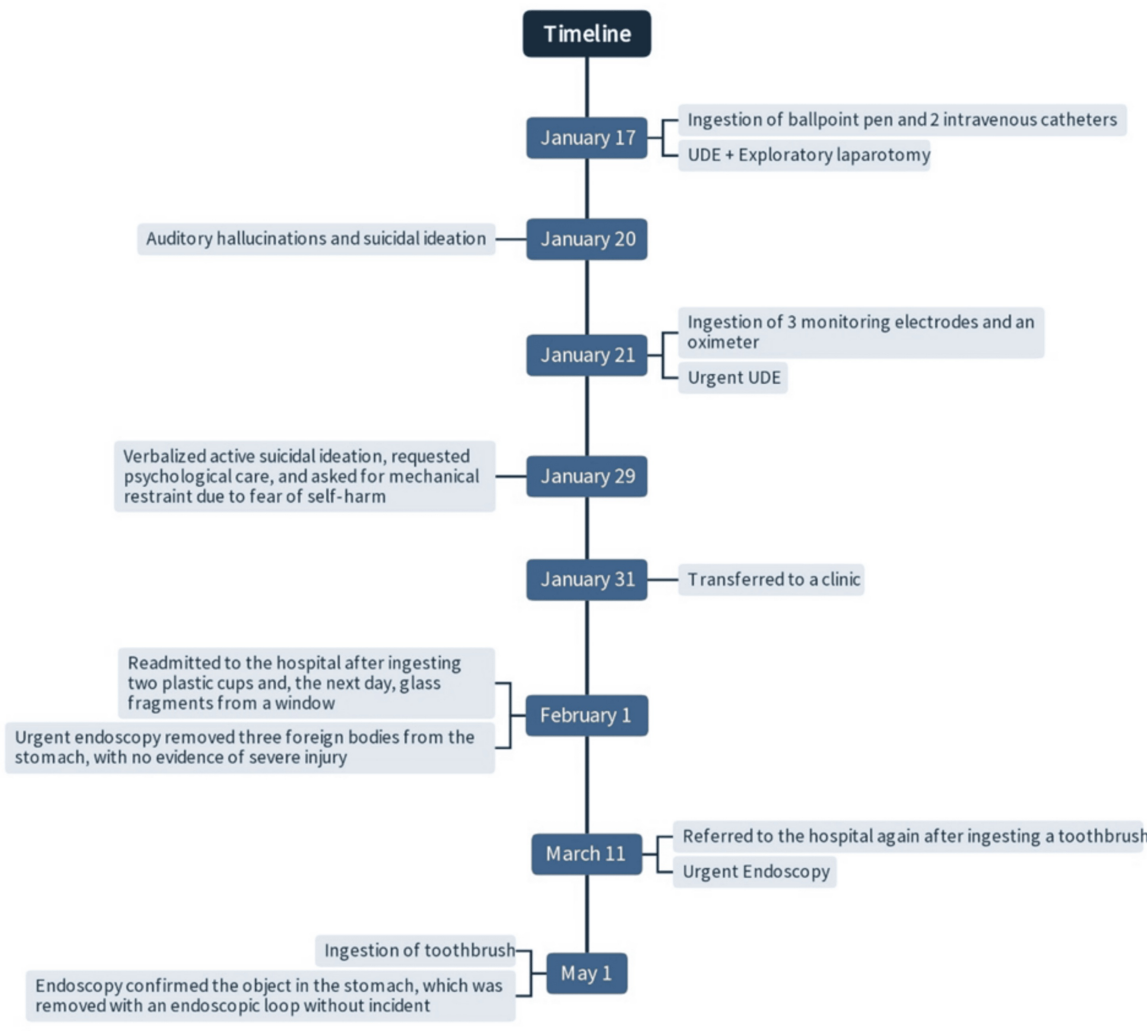
Timeline of clinical events and interventions related to repeated foreign body ingestion and psychiatric episodes. This timeline illustrates the sequence of clinical events involving repeated foreign body ingestion, psychiatric symptoms, and corresponding medical interventions. Key dates include instances of object ingestion, hospital admissions, surgical and endoscopic procedures, and psychiatric evaluations. The chart emphasizes the recurrent nature of self-harming behaviors and the multidisciplinary medical response required for patient management.

The patient experienced multiple subsequent hospitalizations due to recurrent episodes of foreign body ingestion. On May 1, 2025, he was admitted again after ingesting a toothbrush, marking his most recent hospitalization. Endoscopy confirmed the object in the stomach, which was removed with an endoscopic loop without incident. Figure 3 presents the timeline of clinical events and interventions related to repeated foreign body ingestion and psychiatric episodes.

## Discussion

The occurrence of pica behaviors - ingestion of non-food substances - among patients with schizophrenia is relatively common. Although pica is not frequently diagnosed alongside schizophrenia, studies indicate a prevalence rate of 14.3% in the early stages of the disease [6]. This behavior may be associated with multiple factors, including neurobiological changes, nutritional deficiencies, psychiatric comorbidities, side effects of psychotropic medications, and non-adherence to treatment. In the reported case, the patient had a history of schizophrenia with heightened impulsivity, auditory hallucinations, and compulsive ingestion of foreign bodies, which aligns with findings in the literature.

The study by You et al. [7] discusses a similar case in which a decompensated schizophrenic patient exhibited episodes of pica driven by delusions and disorganized thinking [7]. Unlike the traditional hypothesis linking pica to nutritional deficiencies, the study suggests that, in psychotic patients, the ingestion of objects may be directly related to their delusional beliefs and lack of insight into their condition. In this case report, the patient believed that ingesting metal would turn him into a police officer, illustrating how delusional thinking can drive such behavior [7].

Several hypotheses have been proposed to explain the relationship between allotriophagy and schizophrenia. Alterations in the dopaminergic and serotonergic systems, often linked to the pathophysiology of schizophrenia, may lead to behavioral dysregulation, including the ingestion of non-nutritive substances. Additionally, the literature suggests that some antipsychotics, such as olanzapine, may induce compulsive eating behaviors, possibly due to their effects on the cortico-basal ganglia system [6].

There are serious health risks associated with pica, including intestinal obstruction, gastrointestinal perforation, intoxication, and metabolic disorders. Possible causes include the effects of psychotropic medications, malnutrition, comorbidity with obsessive-compulsive disorder, cognitive deficits, and delusional beliefs [6]. The patient in this report required surgical procedures to remove ingested objects, as well as psychiatric interventions to manage impulsivity and the underlying disorder. This scenario reflects the complexity of treating allotriophagy in schizophrenia, necessitating a continuous multidisciplinary approach to minimize risks and prevent further ingestions.

Despite the severity of associated complications, there is a scarcity of clinical trials evaluating the efficacy of specific interventions for pica in patients with schizophrenia. Management typically involves a combination of pharmacotherapy and cognitive-behavioral approaches, though the literature has yet to reach a consensus on the optimal therapeutic strategy [6]. In the presented case, even with medication adjustments and containment measures, the patient continued to experience recurrent episodes of foreign body ingestion, highlighting the treatment challenges and the need for more effective strategies.

In this context, allotriophagy in schizophrenic patients represents a significant clinical challenge, requiring integrated management protocols that combine psychiatric support, continuous monitoring, and preventive interventions. The lack of longitudinal research on this association underscores the need for further studies to better understand the underlying mechanisms and develop more effective therapeutic strategies.

## Conclusions

The described case highlights the complexity of managing schizophrenic patients with allotriophagy, a condition that entails not only physical risks but also significant psychiatric and social challenges. The compulsive ingestion of objects underscores the need for continuous monitoring and integrated therapeutic approaches, combining pharmacological treatment, psychological support, and social interventions. The recurrence of this behavior demonstrates the difficulty in controlling impulsivity and the importance of structured support, including family and institutional assistance.
